# Quinolin-4-ones: Methods of Synthesis and Application in Medicine

**DOI:** 10.3390/molecules30010163

**Published:** 2025-01-03

**Authors:** Katarzyna Gach-Janczak, Justyna Piekielna-Ciesielska, Jakub Waśkiewicz, Kamil Krakowiak, Karol Wtorek, Anna Janecka

**Affiliations:** Department of Biomolecular Chemistry, Medical University of Lodz, Mazowiecka 6/8, 92-215 Lodz, Poland; katarzyna.gach@umed.lodz.pl (K.G.-J.); justyna.piekielna@umed.lodz.pl (J.P.-C.); jakub.waskiewicz@student.umed.lodz.pl (J.W.); kamil.krakowiak@student.umed.lodz.pl (K.K.); karol.wtorek@umed.lodz.pl (K.W.)

**Keywords:** chemical synthesis, antibacterial agents, antiviral agents, anticancer activity, structure–activity relationship

## Abstract

Quinolinones, also called quinolones, are a group of heterocyclic compounds with a broad spectrum of biological activities. These compounds occur naturally in plants and microorganisms but can also be obtained synthetically. The first synthesis of quinolinones took place at the end of the 19th century, and the most recent methods were published just a few years ago. They allow for obtaining an unlimited number of analogs differing in biological properties. In this review, we described the plethora of methods leading to quinolin-4-ones. Several of these compounds have been used as antibiotics for over four decades, but recently, their antiproliferative effects have been of particular interest to researchers. This review summarizes the experimental progress made in the synthetic development of various routes leading to quinoline-4-ones and presents an overview of the structures, their evolution, and their relation to activity.

## 1. Introduction

Heterocycles containing a nitrogen atom in their structure are a very important group of organic compounds that have found numerous applications in organic synthesis, medicinal chemistry, and everyday life. Alkaloids, antibiotics, vitamins, hormones, and a large number of drugs and dyes contain azaheterocyclic ring systems. The properties of azaheterocyclic compounds depend on the specific structure of the heterocyclic ring and the type of functional groups present in the molecule. These compounds often exhibit biological activities such as antibacterial, antifungal, or anticancer [1].

**Figure 1:**
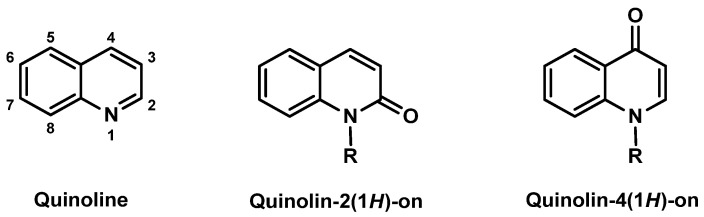
The structure of quinoline and quinolinones.

An interesting group of azaheterocyclic compounds is quinolinones (also called quinolones or azaflavones in some sources). They contain a quinoline ring system linked to a carbonyl group and can be divided into quinolin-2(1*H*)-ones and quinolin-4(1*H*)-ones (Figure 1).

**Figure 2:**
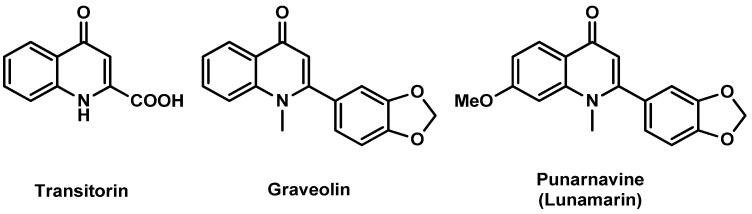
Quinolin-4-ones of plant origin with interesting biological properties.

Quinolinones are relatively rare in nature. The quinolin-4-one skeleton can be found in plants of the *Rutaceae*, *Rubiaceae*, and *Astraceae* families and in microorganisms [2]. Some of the naturally occurring quinolin-4-ones with interesting activities isolated from plants are shown in Figure 2.

**Transitorin** inhibits the growth of bacteria such as *Staphylococcus aureus*, *Pseudomonas aeruginosa*, and Enterobacter cloacae [3]. **Graveolin** has versatile pharmacological activity, including antifungal [4] and antimicrobial [5] properties. Graveolin has also been shown to have cytotoxic activity against A375 cutaneous melanoma cells [6]. Synthetic derivatives of graveolin inhibited the process of angiogenesis during the development of malignant lesions [7].

A derivative of graveolin with a methoxy group in position 7 is **punarnavine** (also called lunamarin). This compound was isolated from the roots of *Boerhaavia diffusa* (family *Nyctaginaceae*), a plant used in Indian folk medicine. Punarnavine was shown to possess various properties, including hepatoprotective [8], immunomodulatory [9], anti-stress [10], and anti-oxidative [11]. Recently, attention has also been drawn to the anticancer effects of punarnavine. In low, non-toxic concentrations, this compound induces apoptosis in B16F-10 mouse melanoma cells [12]. In studies using a mouse model of breast tumor 4T1, which mimics stage four human breast cancer, punarnavine was shown to inhibit primary tumor growth and reduce the progression of metastasis to lymph nodes, liver, and lung. In addition, punarnavine is non-genotoxic [13] and inhibits angiogenesis [14], making it a good candidate for an anticancer drug.

**Figure 3:**
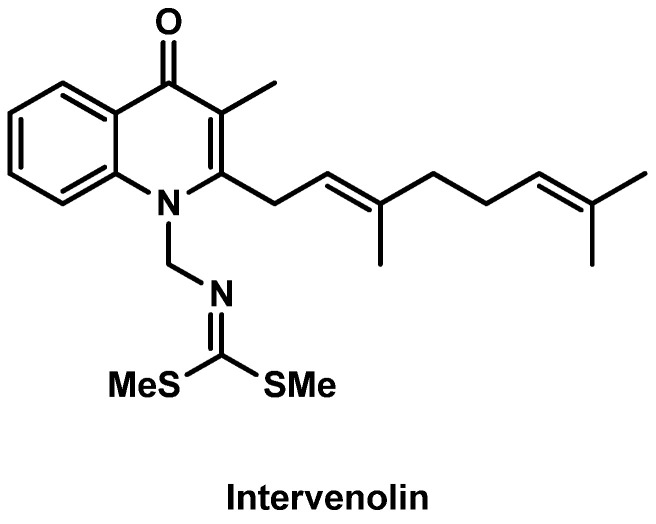
The structure of intervenolin, a quinolin-4-one isolated from microorganisms.

Among quinolin-4-ones of therapeutic importance isolated from microorganisms, the most interesting is **intervenolin** (Figure 3), found in aerobic Gram-positive bacteria of the genus *Nocardia* (family *Nocardiaceae*) [15]. This alkaloid selectively inhibited the proliferation of human gastric cancer cells MKN-74 and MKN-7 and colon cancer cells HTC-15.

Two unique substituents can be distinguished in the structure of this compound: a bis(methylthio)methyleneaminomethyl group on the nitrogen atom and a geranyl chain on the C2, which both have a strong effect on the biological activity of intervenolin [16].

Despite their interesting biological activities, none of the quinoline-4-ones found in nature has been recommended as a drug so far. However, due to the large number of potential functionalizations at different positions, quinoline-4-ones are attractive precursors for the synthesis of biologically important structures, some of which have already become approved medications. Here, we focused on showing various routes leading to synthetic quinoline-4-ones and discussing their medical uses.

## 2. Chemical Methods of Synthesis of Quinolin-4-ones

**Figure 4:**
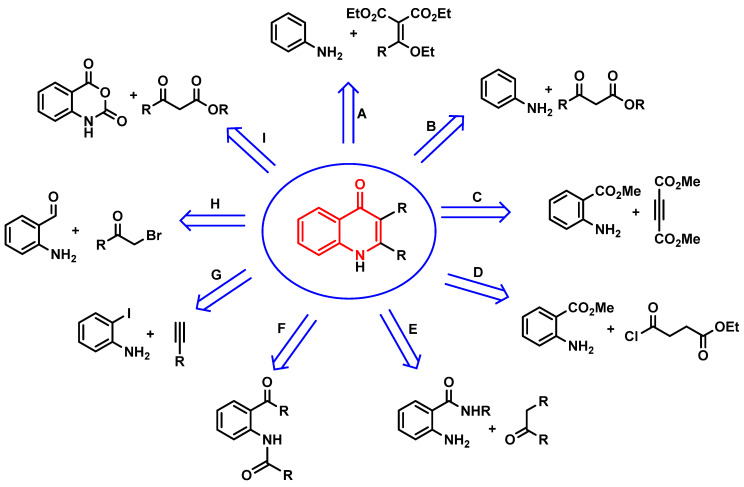
Quinolin-4-one retrosynthesis pathways.

Over the years, many methods have been developed for the synthesis of the quinolin-4-one core. Due to the high possibility of functionalization of the molecule, different strategies and starting materials have been used (Figure 4). These compounds can be obtained with classical methods starting from aniline derivatives, for example, via the Gould-Jacobs (route A) or Conrad-Limpach (route B), as well as starting from methyl anthranilate via Biere-Seelen synthesis (route C) or using Dieckmann condensation (route D). Less common 3-substituted quinolin-4-ones were obtained by the Snieckus reaction from an anthranilic acid amide (route E) or the Camps reaction from *N*-(2-acylaryl)amides (route F). Modern methods include transformations catalyzed with transition metals (route G) or *N*-heterocyclic carbenes (route H). Recently, a method has also been developed for the synthesis of quinolin-4-ones from isatoic anhydride via a decarboxylating cyclization reaction (route I).

Some other important reviews in the field of quinolinone synthesis should be mentioned here. Rao et al. [17] described quinolinone synthesis by electrochemically and photochemically driven methods. Dine et al. [18] provided protocols for metal-free reactions and transition metal-catalyzed procedures. Shen et al. [19] summarized the experimental progress made in the synthetic development of various catalytic routes to preparing quinoline-4-ones and their analogs.

### 2.1. Gould–Jacobs Method (Route A)

**Scheme 1:**
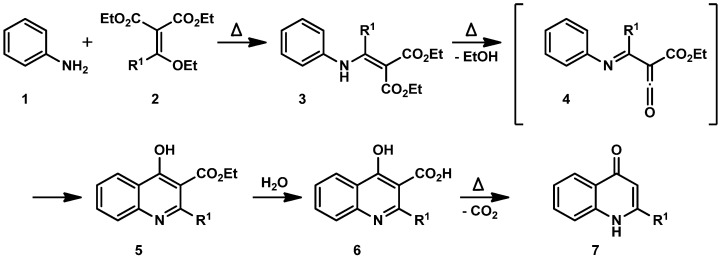
Gould–Jacobs reaction.

The Gould–Jacobs reaction [20] is an important thermal cyclization method leading to the quinolin-4-one backbone. The original approach, proposed in 1939 (Scheme 1), in the first step, involves condensation of aniline **1** and diethyl ethoxymethylidenedimalonate **2**. The resulting diester **3**, upon further heating, is readily cyclized through a ketene intermediate compound **4**, usually under drastic conditions, with the formation of 4-hydroxy-3-ethoxycarbonylquinoline **5**. Hydrolysis of the ester **5** to a carboxylic acid **6** and subsequent decarboxylation yields the corresponding quinolin-4-one **7**. This method allows for the synthesis of many commercially available drugs whose structure is based on the quinoline-4-one backbone, such as the antibiotics nalidixic acid, oxolinic acid, and norfloxacin.

An important aspect is also the regioselectivity of the Gould–Jacobs reaction, which is generally controlled by both steric and electronic factors [21]. Cyclization can occur at either of the two *ortho* positions of aniline, and therefore, when asymmetrically substituted anilines are used, a mixture of products is often obtained.

This classical approach requires a multi-step reaction sequence generally occurring in low overall yields, and temperatures higher than 250 °C are necessary for the cyclization step, which may lead to product decomposition and undesirable side reactions [22].

### 2.2. Conrad–Limpach Method (Pathway B)

**Scheme 2:**
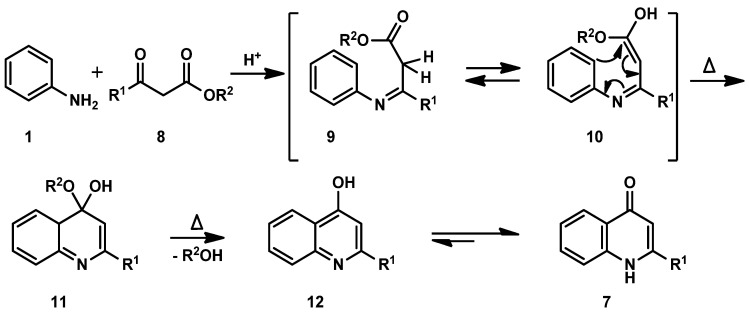
Conrad–Limpach reaction.

The reaction pathway shown in Scheme 2 was developed by Max Conrad and Leonhard Limpach in 1887 [23] and is still successively used [24]. The method involves the condensation of aniline **1** with acetyl acetic ester **8**, with the formation of iminoester **9**, which is in equilibrium with the iminoenol form **10**. The iminoenol **10** then undergoes an intramolecular hetero-Diels–Alder reaction to form hemiketal **11**, which, after alcohol elimination and keto-enol tautomerization, yields quinolin-4-one **7**. Since in this method, the high-energy iminoenol **10** must be indirectly formed, and temporary dearomatization (hemiketal **11**) occurs, the synthesis requires rather drastic conditions (high temperature, strong acid as catalyst). Thus, in early work, cyclization was carried out by heating the Schiff base to high temperatures (200–250 °C) without using a solvent, but the reaction yields were very moderate (less than 30%). When an inert, high-boiling solvent such as mineral oil, diphenyl ether, or Dowtherm A was used, the cyclization yields increased to 95% in many cases [25].

As with the Gould–Jacobs reaction, the presented method has its limitations related to conducting the reaction at very high temperatures. Mineral oil, diphenyl ether, and Dowtherm A, commonly used as solvents, are expensive and difficult to remove from the reaction mixture [26].

### 2.3. Biere-Seelen’s Synthesis (Route C)

**Scheme 3:**
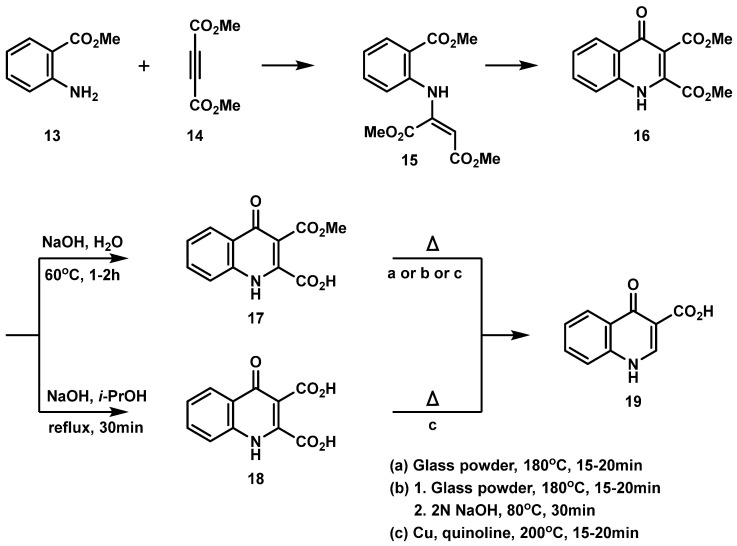
Biere-Seelen’s reaction.

Biere and Seelen’s approach [27] to the synthesis of quinoline-4-ones was developed in 1979 (Scheme 3). The procedure begins with Michael’s addition of dimethyl acetylenedicarboxylate **14** to methyl anthranilate **13**, leading to enaminoester **15**. This ester then undergoes cyclization to quinolin-4-one in the presence of a strong base with the formation of diester **16**. Base hydrolysis using aqueous sodium hydroxide solution occurs regioselectively at position 2 to give ester **17**, while hydrolysis carried out in 2-propanol at boiling temperature leads to the formation of dicarboxylic acid **18**. The final step is the thermal decarboxylation of ester **17** (method a) to produce a mixture of quinolin-3-carboxylic acid **19** and its methyl ester, which are easily separated by column chromatography (14% yield). In contrast, this reaction carried out with the addition of sodium hydroxide solution (method b) yielded only acid **19** with a yield of 92%. The authors also carried out copper-catalyzed thermal decarboxylation of both monoester **17** and dicarboxylic acid **18** (method c), which yielded acid **19** in 52% yield.

The cycloacylation of aniline derivatives to quinoline-4-ones in the presence of Eaton’s reagent was also described [28]. This high-yielding methodology is applicable to a wide variety of functionalized anilines and requires milder conditions than those traditionally employed.

### 2.4. Dieckmann’s Condensation (Route D)

**Scheme 4:**
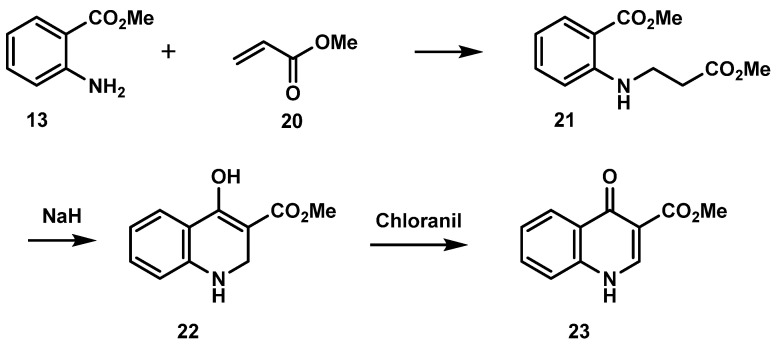
Dieckmann’s reaction.

Dieckmann’s condensation [29,30] is an intramolecular reaction that diesters undergo with the formation of cyclic β-ketoesters. As a result of the reaction of methyl anthranilate **13** with methyl acrylate **20**, a diester **21** can yield, which undergoes intramolecular cyclization in the presence of sodium hydride to give dihydroquinolinone **22** (Scheme 4). Subsequent oxidation with chloranil leads to quinolin-4-one **23**.

### 2.5. Snieckus’ Synthesis (Route E)

**Scheme 5:**
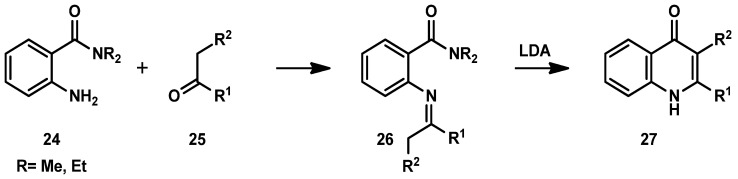
Snieckus’ reaction.

One of the few methods for the synthesis of 3-substituted quinolin-4-ones was developed by the Victor Snieckus group (Scheme 5) [31]. They proposed a regioselective sequence of *ortho*-metallation reactions with subsequent amination, which is a modification of the Niementovsky reaction, allowing for the obtaining of γ-hydroxyquinoline derivatives. Condensation of an anthranilic acid amide **24** with a ketone **25** gave imine **26**, which was difficult to purify, so it was used directly for the subsequent cyclization reaction. Treatment of imine **26** with a strong base (LDA) led to quinolin-4-one **27**. The Snieckus’ method appears to be an interesting route for the synthesis of 3-substituted quinolin-4-ones due to the availability of substituted anthranilic acid amides and the relatively mild conditions of the base-promoted reaction.

### 2.6. Cyclization of N-(2-Acylaryl)amides by Camps’ Method (Route F)

**Scheme 6:**
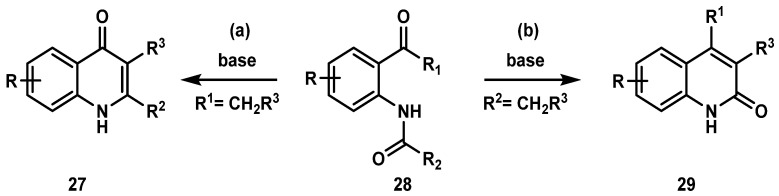
Camps’ cyclization reaction.

In 1899, Rudolf Camps [32,33] presented the synthesis of quinolin-2-ones and quinolin-4-ones by base-catalyzed intramolecular cyclization of *N*-(2-acylaryl)amides **28**. Depending on the structure of the substrate and the conditions used, the reaction leads to the formation of quinolin-4-one **27** (a) or quinolin-2-one **29** (b) (Scheme 6).

**Scheme 7:**
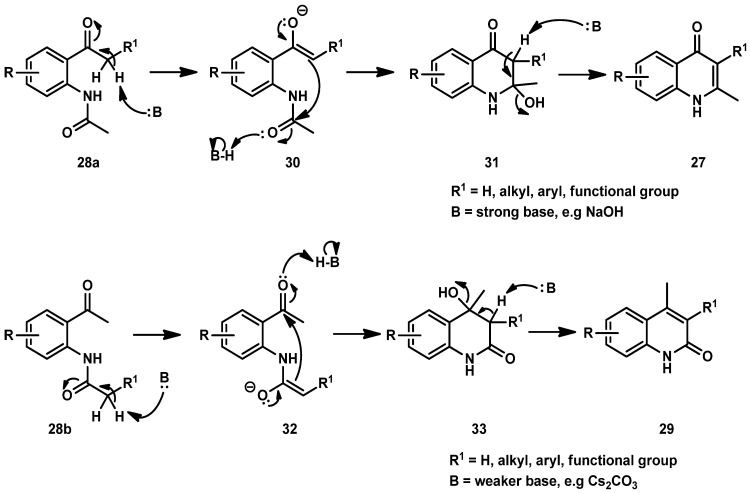
Mechanisms of Camps’ cyclization.

The mechanism of the Camps’ reaction (Scheme 7) involves intramolecular aldol condensation of *N*-(2-acylaryl)amides **28**, which are capable of enolization. When a strong base, such as sodium hydroxide, is used, one of the protons of the CH_2_ group next to the carbonyl is deprotonated, and the formed enolate ion **30** attacks the carbonyl group of the amide function. The formed alcohol **31** then undergoes β-elimination to give quinolin-4-one **7**. The synthesis of quinolin-2-one occurs by deprotonating one of the protons of the CH_2_ group of the amide with a weaker base. The formed enolate ion **32** attacks the carbonyl group of the ketone, and the formed alcohol **33** undergoes elimination to give quinolin-2-one **29**.

**Scheme 8:**
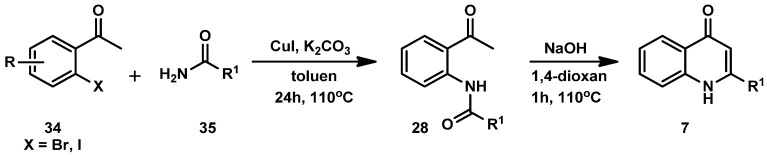
Synthesis of quinoline-4-ones, according to Buchwald.

The Camps’ precursor (**28**) can be easily obtained, for example, by the condensation of 2-aminoacetophenone with acid chloride or the Friedel-Crafts acylation of anilides. In 2007, Buchwald’s group [34] developed a rapid two-step synthesis of 2-aryl- and 2-vinylquinolin-4-ones from *N*-(2-acylaryl)amides **28** using the Camps’ cyclization method (Scheme 8). The precursor **28** was obtained by the copper-catalyzed amidation of 2-halogenoacetophenone **34**. A series of 2-aryl-4-quinolinones **7** was obtained in very good yields (72–97%).

**Scheme 9:**
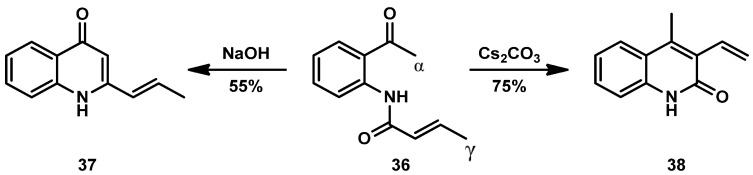
Cyclization of *N*-(2-acetylphenyl)but-2-enamide (**36**) to quinolin-4-one and quinolin-2-one.

The authors also described how the type of base used affects the regioselectivity of the reaction. It was shown that *N*-(2-acetylphenyl)but-2-enamide (**36**) has the ability to cyclize to 2-vinylquinolin-4-one **37** and 4-methyl-3-vinylquinolin-2-one **38** (Scheme 9). In the presence of a stronger base (NaOH), deprotonation occurs at the α position of the ketone, followed by intramolecular aldol condensation with the formation of quinolin-4-one **37**. However, the use of a weaker base (Cs_2_CO_3_) gave quinolin-2-one **38** as the major product via γ deprotonation of the amide. In both cases, trace amounts of the second isomer were formed, which could be easily separated by column chromatography.

### 2.7. Palladium-Catalyzed Carbonylation Reaction (Route G)

Palladium-catalyzed carbonylation reactions are commonly used in the synthesis of quinolin-4-ones [19,35]. These reactions typically use carbon monoxide as the source of the carbonyl group. The coupling reaction of 2-iodoaniline **39** and terminal acetylenes **40** under CO to synthesize quinolin-4-ones was first described by Torii’s group [36]. The reaction used high-pressure CO gas and PdCl_2_(PPh_3_)_2_ or PdCl_2_(dppf)Cl_2_ as a catalyst. The methodology was later improved by Genelot [37,38], who introduced milder reaction conditions.

**Scheme 10:**
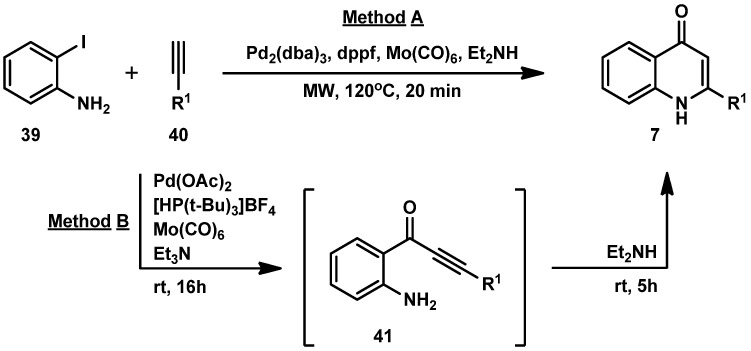
Palladium-catalyzed carbonylation reaction.

In recent years, alternative sources of CO, such as molybdenum or tungsten carbonyls, have been sought as potential replacements for gaseous CO. One example is the use of molybdenum hexacarbonyl [Mo(CO)_6_] in the synthesis of quinolin-4-ones from 2-iodoaniline **39** and terminal acetylenes **40** [39]. This paper describes two protocols that yielded a wide range of 2-substituted quinolin-4-ones **7** with good yields. The first protocol allows the synthesis of the expected compounds as early as after 20 min of microwave (MW) heating (Scheme 10, Method A), while the second procedure is a two-step approach in a single vessel, in which the reaction is carried out at room temperature and functional groups sensitive to Mo(CO)_6_, such as nitro groups, may be present (Scheme 10, Method B).

**Scheme 11:**
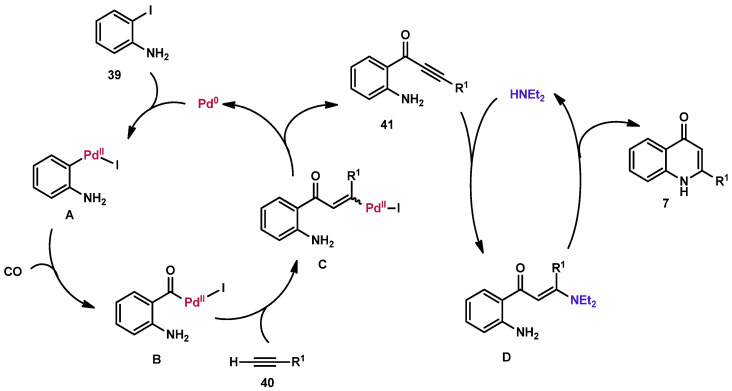
Mechanism of palladium-catalyzed carbonylation reaction.

The reaction mechanism involves Sonogashira carbonylation, resulting in the formation of alkyne **41**, which then undergoes cyclization upon the addition of diethylamine (Scheme 11) [40]. Molybdenum hexacarbonyl decomposes under heating, releasing CO, which then participates in the carbonylation step. As a result of the oxidative addition of the catalyst Pd^0^ to 2-iodoaniline **39**, complex A is formed. In the next step, there is an insertion-1,1 of CO into the palladium–carbon bond of complex A, making complex B susceptible to attack by the nucleophile alkyne **40**. As a result, intermediate complex C is formed, and the carbonylation product **41** is formed by β-elimination and restoration of catalytically active forms of Pd^0^. The addition of diethylamine to the resulting alkyne **53** leads to the formation of intermediate compound D, which undergoes cyclization to give the desired 2-substituted quinolin-4-one **7**.

The use of molybdenum hexacarbonyl as a source of CO has many advantages over traditional methods using gaseous CO. Mo(CO)_6_ is solid, making it easier to use and transport this reactant. In addition, the lower risk of explosion improves safety and comfort. The reagent is relatively inexpensive and readily available, making it an attractive alternative to gaseous CO in large-scale industrial processes.

### 2.8. Environmentally Friendly Syntheses of Quinolin-4-ones

As described above, classical methods are very useful for developing an infinite variety of quinoline-4-one analogs. The drawbacks of these procedures are the use of hazardous chemicals, high temperatures, organic solvents, and long reaction times. All these factors have an impact on the environment and the economy. It is believed that the development of so-called green syntheses, which are alternative technologies seeking replacements for hazardous substances and trying to reduce waste and conserve energy, is the future of organic chemistry. Also, in the field of heterocyclic compounds, efforts are made to develop new, environmentally friendly procedures [41].

#### 2.8.1. *N*-Heterocyclic Carbenes-Catalyzed Synthesis of Quinolin-4-ones (Route H)

**Scheme 12:**
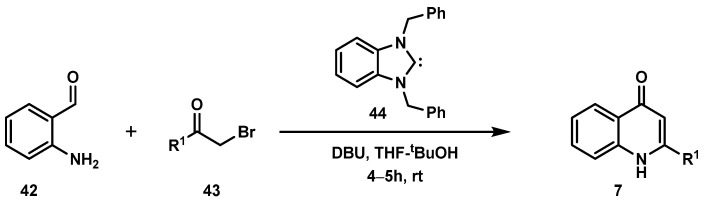
NHC-catalyzed synthesis of quinoline-4-ones.

*N*-heterocyclic carbenes (NHCs) are electron-rich chemical entities that exhibit a nucleophilic character [42]. They play an important role in modern organic chemistry. They are mainly used in reactions catalyzed by organometallic compounds and in organocatalysis. NHC-catalyzed synthesis of quinolin-4-ones has been described, among others, by Rai and co-workers [43]. The authors developed a method to obtain products in high yields by dehydrative acetylation of 2 aminobenzaldehyde **42** with α-halogenketone **43** and subsequent intramolecular heterocyclization (Scheme 12). Due to the mild reaction conditions, simplicity, and lack of by-products, this approach is an interesting alternative to already existing procedures for obtaining quinolin-4-ones **7**.

**Scheme 13:**
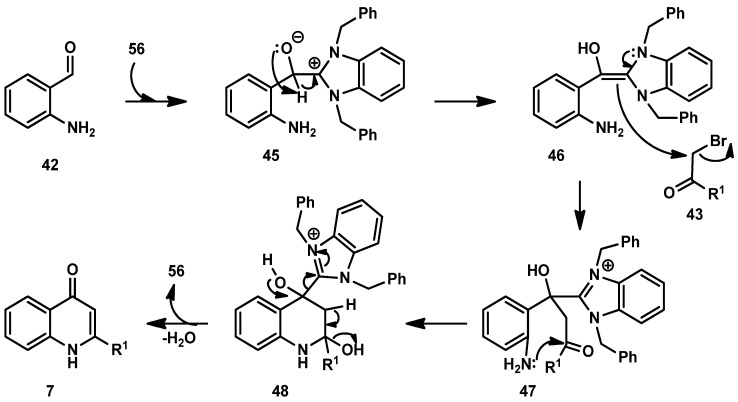
Mechanism of NHC-catalyzed synthesis of quinolin-4-ones.

Scheme 13 shows the proposed reaction mechanism. The nucleophilic attack of the carbene **44** on the carbonyl carbon of the aldehyde **42** leads to the formation of the intermediate compound **45**. In the second step, a proton transfer occurs in **45** to produce homoenolate **46**. Nucleophilic homoenolate **46** attacks bromide **43** to give adduct **47**, which undergoes intramolecular heterocyclization. The resulting adduct **48** undergoes dehydration, yielding product **7**. The increased stability of the conjugated quinolin-4-one **7** double bonds is probably the driving force of the dehydration reaction.

#### 2.8.2. Decarboxylating Cyclization (Route I)

**Scheme 14:**
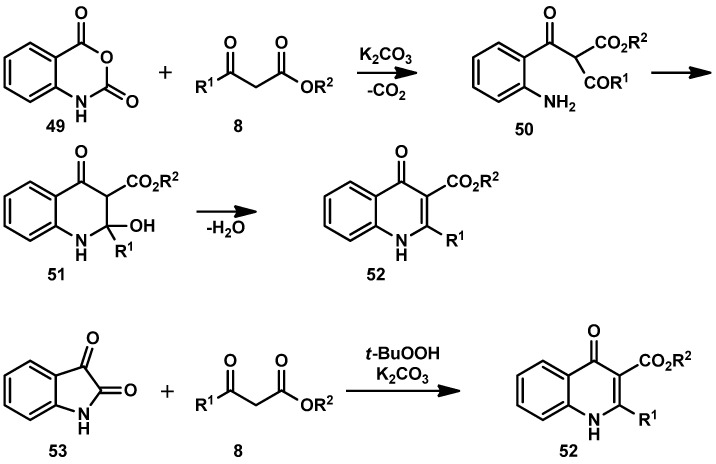
Mechanism of NHC-catalyzed synthesis of quinolin-4-ones.

Recently, Sun and co-workers [44] developed an environmentally friendly decarboxylating cyclization procedure to synthesize quinolin-4-ones (Scheme 14). The starting materials are inexpensive and readily available isatoic anhydrides **49** and 1,3 dicarbonyl compounds **8**. The base generates a carbanion from compound **8**, which attacks the carbonyl group of anhydride **49** to give intermediate **50** with the simultaneous release of CO_2_. Following intramolecular cyclization and dehydration, a suitably substituted quinolin-4-one **52** is formed. The reaction was carried out in water at 80 °C. The reaction was also performed using isatin **53** as a substrate in dimethyl sulfoxide (DMSO). The added oxidant, *tert*-butyl hydroperoxide, converted isatin **53** to isatoic anhydride **49**, and the subsequent addition of 1,3-dicarbonyl compound **8** gave the desired quinolin-4-one **52**. Thanks to the mild reaction conditions, the synthesis is environmentally friendly, and the only by-products are carbon dioxide and water. No toxic transition metals are used in the reaction, while a wide range of products can also be obtained on a large scale.

## 3. Quinolin-4-ones as Drugs

Quinolin-4-ones and their derivatives are important organic compounds because of their wide range of biological activities, which makes them attractive therapeutic agents [45]. Seventeen compounds containing a quinolin-4-one motif have been marketed to date, according to the Drug Bank Online database [46]. The majority of these drugs are antibiotics from the fluoroquinolinone group (norfloxacin, ciprofloxacin, oflokxacin, perflofloxacin, lomefloxacin, levofloxacin, grepafloxacin, sparfloxacin, moxifloxacin, besifloxacin, rosoxacin, delafloxacin, finafloxacin, gatifloxacin, but also antiviral drugs (elvitegravir) and those used to treat asthma (nedocromil) and cystic fibrosis (ivacaftor).

The quinolinone scaffold is also incorporated into compounds that display anti-malarial activity, such as endochin and its derivatives and analogs [47,48]. However, reports on the anti-malarial properties of quinolinones, compared to their antibacterial properties, are relatively limited and will not be discussed in this review.

### 3.1. Antibacterial Agents

Quinolones are one of the most important families of antimicrobial agents, representing fully synthetic compounds, as opposed to antibiotics, which are produced by microorganisms [2].

#### 3.1.1. Four Generations of Quinolinones

**Figure 5:**
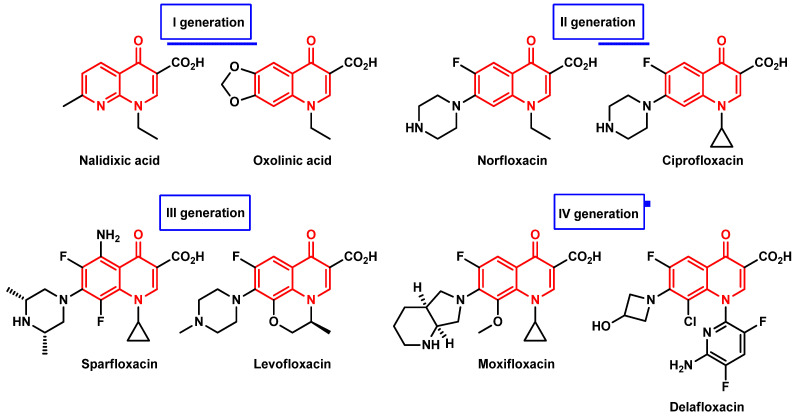
Examples of the I-st, II-nd, III-rd, and IV-th generation quinoline-4-ones.

First-generation quinoline-4-ones, nalidixic acid (technically a naphthyridone) first synthesized by Lesher [49], and oxolinic acid [50] developed in Japan in the 1970s (Figure 5) were mainly used in the treatment of urinary tract infections, but their use was limited due to the narrow spectrum of action (some Gram-negative bacteria), short half-life, and a number of side effects, and nowadays these agents are not recommended for therapy [51].

Soon, new analogs with better properties, modified primarily at the nitrogen atom and at the C-6, C-7, and C-8 positions, were synthesized. In the late 1970s, there was a breakthrough in the development of quinolinone-based drugs with the introduction of the fluorine atom into the quinolin-4-one skeleton in position 6 [52,53,54]. This modification significantly improved the spectrum of activity and yielded the second-generation quinoline-4-ones. The first approved drug from this group was norfloxacin, which showed enhanced antimicrobial activity against Gram-negative and some Gram-positive bacteria and reduced toxicity [55,56,57] (Figure 5).

The third-generation quinoline-4-ones have expanded activity against Gram-positive and Gram-negative bacteria. The fourth-generation quinolinones retain the Gram-negative and Gram-positive antimicrobial activity and inhibit anaerobic bacteria [58].

Almost all of the second-, third-, and fourth-generation quinoline-4-ones possess a fluorine atom at position 6, with the exception of the most recent compounds from the fourth generation. Although the fluorine at C6 significantly improves activity, current research is focused on its removal because it is related to genotoxicity. The reduction in potency caused by its removal can be compensated for by using alternative substituents at other positions [59].

Some important antibacterials from the group of fluoroquinolin-4-ones are norfloxacin, ciprofloxacin, levofloxacin, and moxifloxacin.

**Norfloxacin** is not only the first synthesized fluoroquinolin-4-one derivative but is also the first quinolinone drug approved for use that contains a piperazine group. Norfloxacin is highly active against most Gram-positive and Gram-negative bacteria. It was patented in 1979 by Kyorin [60] and four years later approved for clinical use.

Another example of a second-generation quinolin-4-one and the best-known antibacterial of the fluoroquinolinone group is **ciprofloxacin**. It is a drug used for a wide range of bacterial infections when other antibiotics have failed. In the early 2000s, ciprofloxacin was also used to treat patients after inhalation exposure to anthrax bacillus after bioterrorist attacks in the United States. Ciprofloxacin was first synthesized by Klaus Grohe and patented by Bayer in 1983 [61].

**Ofloxacin** is a racemic second-generation quinolin-4-one. Its (*S*)-isomer, levofloxacin (a third-generation quinolin-4-one), is the first known optically active fluoroquinolinone. Both compounds were developed by Daiichi Pharmaceuticals in 1985 and 1993, respectively, and both have broad-spectrum antimicrobial activity. Levofloxacin additionally acts against penicillin-resistant *Streptococcus pneumoniae*, the pneumonia germ responsible for the development of respiratory tract infections, sinusitis, pneumonia, meningitis, and sepsis.

The important fourth-generation quinoline-4-one is **moxifloxacin**. It was developed by Bayer AG (Leverkusen, Germany) in 1989. It is used in the treatment of a wide range of infections caused by Gram-positive and Gram-negative bacteria and other microorganisms (*Chlamydia trachomatis*). Like other fluoroquinolinones, moxifloxacin easily penetrates into tissues and body fluids. It is used to treat many infections, including cellulitis, anthrax, and abdominal and respiratory tract infections. Drugs containing moxifloxacin were introduced to the market in 1999 and are currently sold in more than 80 countries around the world.

#### 3.1.2. Mechanism of Antimicrobial Action of Quinolinones

The mechanism of action of fluoroquinolin-4-ones is interference with nucleic acid synthesis. They inhibit bacterial DNA synthesis by disrupting the enzymes topoisomerase IV and DNA gyrase, resulting in rapid bacterial death [62,63]. As a general rule, Gram-negative bacterial activity correlates with the inhibition of DNA gyrase, and Gram-positive activity corresponds with the inhibition of DNA type IV topoisomerase. First- and second-generation fluoroquinolinones bind either to DNA gyrase or to DNA topoisomerase IV, depending on the bacteria and the drug, whereas newer fluoroquinolin-4-ones may target both of these enzymes with equal affinity in any organism [2].

The broad spectrum of activity and excellent tissue penetration make fluoroquinolin-4-ones potent antibacterial drugs, but an increasing number of resistant pathogens becomes a factor limiting their use [51,64,65,66].

#### 3.1.3. Structure–Antibacterial Activity of Fluoroquinolin-4-ones

**Figure 6:**
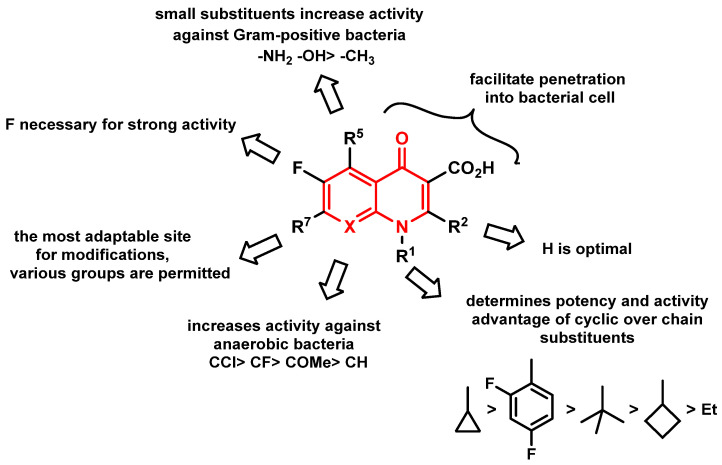
Relationship between structure and antibacterial activity of fluoroquinolin-4-ones.

Many analyses have been carried out to determine the relationship between the structure and antibacterial activity of fluoroquinolin-4-ones [52,59] (Figure 6). Substituents placed on the N1 nitrogen atom are essential for this activity. The cyclopropyl group is considered the best choice (e.g., ciprofloxacin, moxifloxacin). Antibacterial activity of fluoroquinolinones substituted at N1 may result from the steric hindrance of the substituent and also from other spatial effects that affect the molecule’s interaction with the DNA gyrase. The carboxyl group on the C3 and the ketone group in position 4 facilitate penetration of the drug into the bacterial cell. In the C5 position, the introduction of small substituents, such as an amine group (e.g., sparfloxacin) or a methyl group (e.g., grepafloxacin), increases activity, especially against Gram-positive bacteria. In the case of larger substituents, the antimicrobial activity drops drastically. The fluorine atom in position 6 is necessary for strong activity. Substitution at the C7 enhances antimicrobial activity. It is the most adaptable site for modifications. Various groups are permitted, but the optimal substituents are 5- and 6-membered *N*-heterocycles [53]. The introduction of a pyrrolidine ring into this position (e.g., moxifloxacin) results in increased activity against Gram-positive bacteria, while the introduction of piperazine (e.g., ciprofloxacin) is against Gram-negative bacteria. Further substitution of both ring types with an alkyl group increases the solubility of the molecule in water [54]. However, a substituent at the C7 position can be responsible for central nervous system (CNS) side effects and for interactions with non-steroidal anti-inflammatory drugs (NSAIDs) [51]. Fluoroquinolinones with pyrrolidine or piperazine rings containing alkyl groups (e.g., grepafloxacin) cause minimal interactions with other drugs and a lower risk of CNS disorders compared to non-alkylated structures (e.g., ciprofloxacin). Substituents on the C8 improve efficacy against anaerobic bacteria. Although the presence of a halogen at this position (e.g., sparfloxacin) is associated with an expanded spectrum of action against anaerobic bacteria, improved oral absorption, and water solubility, it is, unfortunately, also a major cause of phototoxicity. The presence of a methoxy group at the C8 prevents the phototoxicity of fluoroquinolin-4-ones and may reduce the risk of drug resistance development [65].

### 3.2. Cystic Fibrosis Drugs

**Figure 7:**
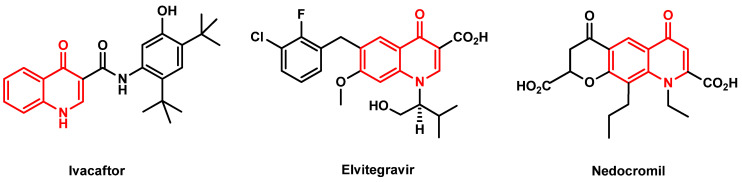
Structure of quinoline-4-one drugs approved for the treatment of cystic fibrosis, HIV, and asthma.

In addition to broad antimicrobial activity, quinolin-4-ones also exhibit other biological properties. **Ivacaftor** (Figure 7) is a drug designed to treat patients with cystic fibrosis. Cystic fibrosis is an incurable inherited disease caused by mutations in the CFTR gene. This gene provides instructions for making a protein called the CF transmembrane conductance regulator, which functions as a channel across the membrane of cells that produce mucus, sweat, saliva, tears, and digestive enzymes. Mutations in the CFTR gene cause clinical symptoms in many areas of the body: the lungs, sinuses, gastrointestinal tract, and reproductive tract.

There are an estimated 100,000 people with cystic fibrosis worldwide. The median survival 50 years ago was 10 years; with today’s medications, patients live up to 50 years. In 2012, Vertex Pharmaceuticals launched a quinolin-4-one derivative, ivacaftor, approved by the FDA to treat cystic fibrosis. This is the first drug that treats the underlying cause rather than the symptoms of the disease. Ivacaftor improves the transport of chloride ions through the ion channels by binding to the channels directly and inducing a non-conventional mode of gating, which in turn increases the probability that the defective channel is open [67,68,69].

### 3.3. Antiviral Drugs

**Elvitegravir** (Figure 7) was the first quinoline-4-one drug developed for the treatment of HIV infection. Its mechanism of action involves the inhibition of HIV integrase, the enzyme essential for the virus to integrate its genetic code into the host’s DNA. Elvitegravir was developed by the pharmaceutical company Gilead Sciences, which licensed the drug from Japan Tobacco in 2008, and it was approved by the FDA for clinical use in 2014 [70,71,72,73].

### 3.4. Antiallergic Drugs

**Nedocromil** (Figure 7) is a quinolinone-based medication whose action is to prevent shortness of breath, wheezing, and other breathing problems caused by asthma. Nedocromil acts as a mast cell stabilizer, inhibits the degranulation of mast cells, and prevents the release of histamine and tryptase, preventing the synthesis of prostaglandins and leukotrienes [74].

## 4. Anticancer Activity of Quinolin-4-ones

According to the World Health Organization (WHO), cancer is one of the most common causes of death before the age of 70, and it is a major global health problem [75]. The search for new anticancer drug candidates is one of the very important goals of medicinal chemistry.

The high costs of obtaining new drugs encourage the search for anticancer agents among already available drugs or their derivatives [76,77]. Quinolin-4-ones possess many desirable features, such as low toxicity, favorable physicochemical properties, a pharmacokinetic profile, and a low incidence of resistance development. The simplicity of the synthesis of these compounds and the large number of potential functionalization sites have made them attractive precursors in the search for new anticancer drug candidates. Many antibiotics from the fluoroquinolinone group, which have been known for years, are now being investigated for their potential anticancer activity [78,79,80,81].

### 4.1. Quinolin-4-one Antibiotics with Anticancer Potential

**Ciprofloxacin** has been shown to induce apoptosis in a variety of cancer cell lines, including lung or breast cancer cells [82,83,84,85]. Various ciprofloxacin derivatives with anticancer properties are described in detail by Nowakowska et al. [86].

**Lomefloxacin** shows high cytotoxic activity against COLO829 melanoma cells [87,88].

**Norfloxacin** analogs were potent against the HeLa cell line with 100% inhibition of cell viability [89].

**Levofloxacin** significantly altered gene expression in a direction favoring anticancer activity. It seems to be an attractive candidate for breast cancer treatment. It inhibited proliferation and induced apoptosis in a panel of breast cancer cell lines while sparing normal breast cells [90,91].

**Figure 8:**
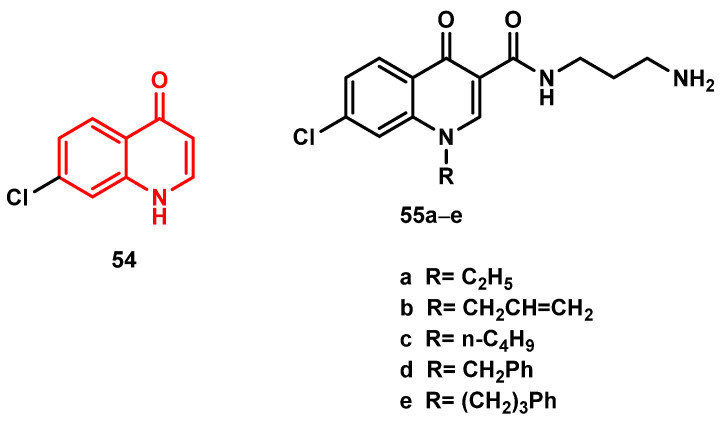
Structure of 7-chloroquinolin-4-one and its 3-amido derivatives **55a**–**e**.

In 2003, China’s State Food and Drug Administration (SFDA) approved **7-chloroquinolin-4-on** (**54**) for the treatment of breast cancer and non-small-cell lung cancer (NSCLC). Its antitumor activity involves blocking DNA synthesis and damaging the tumor cell’s DNA matrix. However, the usefulness of this drug is limited due to its poor solubility. Zhou’s group [92] synthesized derivatives of this compound, obtaining a series of five 3-amido-7-chloroquinolin-4-ones **55a**–**e** with an amide side chain in position 3 and alkyl substituents on the nitrogen atom (Figure 8).

These compounds exhibited a more potent antiproliferative effect against a panel of human tumor cell lines than the lead structure **54**. The best results were obtained for compound **55e**, which showed the most potent antiproliferative activity and exhibited selective cytotoxicity against HepG2 cells.

In the search for more efficient anticancer candidates, the hybridization of various quinolinones with other anticancer pharmacophores has also been investigated [93]. However, up to date, none of the quinoline-4-ones has been approved in Europe or the US for the treatment of cancer.

### 4.2. Mode of Action of Quinolinones as Anticancer Agents

Data indicate that fluoroquinol-4-ones inhibit various proteins and enzymes involved in cancer cell growth, such as topoisomerase II, protein kinases, phosphoinositide 3-kinases (PI3K), and histone deacetylase (HDAC) [94,95,96,97,98]. Such inhibition leads to cell death by apoptosis and cell cycle arrest in the S and G2/M phases [99]. Furthermore, dysfunction of mitochondria, likely due to the similarity between mitochondrial and bacterial cell structures, has been shown as a mechanism of action [97]. The effects of fluoroquinolinones on migration and metastasis have also been reported. In particular, they could suppress matrix metalloproteinase 9 (MMP9) production [100,101,102,103]. Additionally, some quinoline-4-ones were found to be potent inhibitors of tubulin polymerization with high selectivity against tumor cells. 2-Phenylquinolin-4-one displayed an antiproliferative effect in several cancer types through inhibition of tubulin polymerization [104]. Fluoroquinolinones can also hamper cancer growth by indirect effects via modulation of the immune response [105].

### 4.3. Structure–Anticancer Activity of Quinoline-4-ones

Structure–activity relationship studies performed in many laboratories all over the world allowed us to determine which positions in the quinoline ring and which substituents are the most important for anticancer activity [106,107].

**Figure 9:**
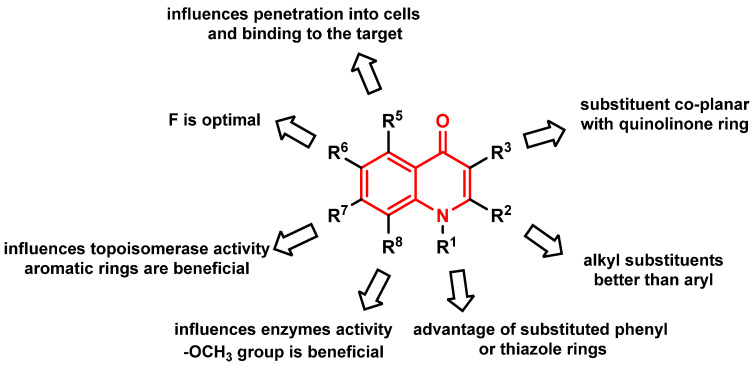
Relationship between structure and anticancer activity of quinolin-4-ones.

Figure 9 summarizes the effects of various substituents and their positions on the biological activity of quinoline-4-ones. A substitution on the nitrogen atom, as well as the presence of a carbonyl group at position 4, are essential for overall potency [78,81,92]. A cyclopropyl group on N1 was found to increase the activity compared to the ethyl group. Substituted phenyl or thiazole rings in this position also have a beneficial effect [81]. Alkyl groups in position 2 turned out to be more advantageous for antineoplastic activity than aryl groups [108]. It is believed that the substituent in position 3 should be coplanar with the quinoline ring [109]. The nature of substituents at C5 can affect the molecule’s penetration into cells and binding to the target. The optimal substituent at position 6 is a fluorine atom [86,89]. The substituent at the C7 carbon is responsible for direct interaction with topoisomerase II in a complex with DNA. The introduction of aromatic rings at this position improves antitumor properties, as does the methoxy group at the C8 carbon atom [89].

## 5. Conclusions

The scaffold of quinolinones is found in nature and has been used for the development of new, biologically active substances. Among many possible isomeric forms, the most common are quinolin-4-ones, which are present in several natural structures but mostly in fully synthetic compounds. Several quinoline-4-ones have been approved as antimicrobial agents and turned out to be invaluable in treating various bacterial infections.

More recently, it has been demonstrated that some quinoline-4-ones exhibit cytotoxic activity against various cancer cell lines and show excellent bioavailability as compared to the current therapeutic drugs for cancer treatment. These properties make them promising candidates for new anticancer agents. The high costs of designing, synthesizing, and marketing new medications encourage the search for anticancer candidates among already available drugs. Therefore, numerous synthetic modifications of existing and approved for clinical use quinoline-4-ones are being conducted, providing more detailed structure–activity relationship studies that may lead to more active moieties. Advances in developing computer-aided models to predict desired features of drugs may be invaluable in designing new generations of quinoline-4-ones. The future directions should be focused on increasing efficacy and reducing undesired side effects and off-target interactions.

## Data Availability

Not applicable.
